# High-end exposure relationships of volatile air toxics and carbon monoxide to community-scale air monitoring stations in Atlanta, Chicago, and Houston

**DOI:** 10.1007/s11869-015-0345-4

**Published:** 2015-04-29

**Authors:** Eric M. Fujita, Barbara Zielinska, David E. Campbell, John C. Sagebiel, Will Ollison

**Affiliations:** Desert Research Institute, 2215 Raggio Parkway, Reno, NV 89512 USA; University of Nevada, Reno, NV 89512 USA; American Petroleum Institute, Washington, DC USA

**Keywords:** Mobile source air toxics, Microenvironment, Exposure, VOC, BTEX, Evaporative emissions

## Abstract

Evaporative and exhaust mobile source air toxic (MSAT) emissions of total volatile organic compounds, carbon monoxide, BTEX (benzene, toluene, ethylbenzene, and xylenes), formaldehyde, acetaldehyde, butadiene, methyl tertiary butyl ether, and ethanol were measured in vehicle-related high-end microenvironments (ME) under worst-case conditions plausibly simulating the >99th percentile of inhalation exposure concentrations in Atlanta (baseline gasoline), Chicago (ethanol-oxygenated gasoline), and Houston (methyl tertiary butyl either-oxygenated gasoline) during winter and summer seasons. High-end MSAT values as ratios of the corresponding measurements at nearby air monitoring stations exceeded the microenvironmental proximity factors used in regulatory exposure models, especially for refueling operations and MEs under reduced ventilation. MSAT concentrations were apportioned between exhaust and evaporative vehicle emissions in Houston where methyl tertiary butyl ether could be used as a vehicle emission tracer. With the exception of vehicle refueling operations, the results indicate that evaporative emissions are a minor component of high-end MSAT exposure concentrations.

## Introduction

Microenvironments (ME) in close proximity to vehicular emissions are typically the largest source of exposure to volatile organic compounds (VOC), fine particulate matter (PM_2.5_), and ultrafine particles (UFP) in urban areas (Westerdahl et al. 2005; Fujita et al. 2007, 2011; Özkaynak et al. 2008). Since traffic-related pollutants disperse rapidly (Zhu et al. 2002), creating sharp gradients in pollutant concentrations near major roadways, pollutant concentrations on and near roadways can be substantially higher than indicated by data from neighborhood-scale air quality monitoring sites (Fujita et al. 2013, 2014). The USEPA regulatory population-based human exposure models, such as the Hazardous Air Pollution Exposure Model (HAPEM) (Rosenbaum and Huang 2007) and the Air Pollution Exposure model (APEX) (US EPA 2008), account for the differences in ambient concentrations measured at a fixed site monitor and the specific MEs by applying scaling factors for different categories of MEs. This approach assumes that pollutant concentrations at MEs are linearly related to the neighborhood-scale ambient measurements by constant multiplicative factors, which is called the proximity factor. This factor is an estimate of the ratio of the outdoor concentration in the immediate vicinity of the ME to the outdoor concentration represented by a nearby neighborhood- or regional-scale air quality data. This simplifying assumption is based on the observations that the relationship between ME concentration and ambient concentration tends to be more linear as averaging times increase (Dockery and Spengler 1981). Recent studies have demonstrated the limitations of relying alone on central-site ambient data and the importance of applying exposure modeling methods incorporating time-activity, home-work/school commuting, and indoor/near-road/outdoor exposure factor data for estimating population pollutant exposures in health studies (Özkaynak et al. 2008, 2013; Sarnat et al. 2013; Baxter et al. 2013).

The Clean Air Act Section 211(b) Tier 2 High-End Exposure Study of Conventional and Oxygenated Gasoline was conducted to measure concentrations of volatile vehicular evaporative and exhaust mobile source air toxic (MSAT) emissions in microenvironments under conditions that characterize the plausible upper 1 % of inhalation exposure concentrations (Zielinska et al. 2003, 2009). The Desert Research Institute (DRI) and Southwest Research Institute (SwRI) conducted controlled measurements of relationships between vehicle exhaust emission rates and pollutant concentrations inside a trailing vehicle and an attached residential garage (Zielinska et al. 2012) as well as urban microenvironments at potentially high-end exposure locations and conditions in Atlanta, Chicago, and Houston during winter and summer seasons (Zielinska et al. 2013). Target species included total volatile organic compounds (TVOC), carbon monoxide (CO), benzene, toluene, ethylbenzene, and xylenes (BTEX), formaldehyde (HCHO), acetaldehyde (CH_3_CHO), butadiene (1,3-BD), methyl tertiary butyl ether (MTBE), and ethanol (EtOH).

Since only high-end conditions were sampled, the precise percentile range of high-end values cannot be determined from the data collected. Discussions with EPA staff during protocol development lead to the decision to characterize “high-end” exposure concentrations as ≥99th percentile. This rationale followed from the compounding effect of multiple independent selection criteria used to identify high-end conditions. For example, if a single sampling criterion led to exposure concentrations at the 80th or 90th percentile, then samples that met several such criteria should plausibly exceed the 99th percentile when compounded. This is a conservative estimate since a number of individual sampling criteria (e.g., low to calm wind speeds, down-wind locations, enclosed cold start situations, congested rush-hour conditions, scripted fuel spillage during refueling) could by themselves on occasion change near-source exposure concentrations 100-fold from their opposite extremes.

The high-end MSAT and CO concentrations measured within the microenvironments in close proximity to gasoline motor vehicles were previously summarized by Zielinska et al. (2012)). This article relates the ME concentrations to the corresponding measurements at nearby air monitoring stations and adds to the available set real-world measurements of gaseous pollutant proximity factors for high-end urban exposure MEs. Experiments were also conducted to determine the effect of ventilation condition, proximity, and emission levels of a leading vehicle on in-vehicle exposure concentrations within a trailing vehicle. Additionally, the MSAT exposure concentrations in Houston were apportioned between exhaust and evaporative vehicle emissions based upon the ambient microenvironment MTBE/benzene ratios relative to the corresponding ratios in fuel headspace and liquid fuel. Contributions from evaporative vehicle emissions were expected to be high in two microenvironments, (1) during refueling and (2) in underground garages. This apportionment was only possible in Houston since nonoxygenated conventional gasoline was used in Atlanta and ethanol oxygenated gasoline in Chicago during our sampling period.

## Experimental

### Microenvironment measurements

DRI measured seasonal in-vehicle exposure concentrations on urban roadways and in other high-end MEs in Houston (6/3 to 7/9/04; 2/1 to 2/8/05), Chicago (8/5 to 8/20/03; 3/3 to 3/17/04), and Atlanta (7/28 to 8/8/02; 8/23 to 9/3/03; 2/10 to 2/29/04) using a combination of time-integrated samples and continuous measurements for the 13 MEs listed in Table 1. Three-to-five replicate 20–40 min measurements were taken with five replicate tests focused on MEs with potentially greater variability in pollutant concentrations. Measurements included time-integrated (20–40 min), short-term (5 min), and continuous (1 min) measurements methods. Integrated samples used whole-air canister samples (CO, BTEX, MTBE, 1,3-BD), acidified 2,4-diphenylhydrazine (DNPH) cartridges (HCHO, CH_3_CHO), and a multi-bed (TenaxTA-Carbotrap-Carbosieve) solid adsorbent tube (EtOH). Short-term measurements used whole-air canisters and solid phase microextraction (SPME) fiber samples. Continuous measurements used both active nondispersive infrared (NDIR) and passive electrochemical (Langan T-15) devices for CO and an active photo-ionization detector (PID) sensing TVOC compounds with ionization potentials below 10.6 eV. Sampling criteria used to identify high-end MEs locations and conditions, and the specific analytical instruments employed are described elsewhere (Zielinska et al. 2012).

**Table 1:** Summary of sample collections in urban microenvironments

ME #	ME description	Replicates	Sampling time (min)
1	In-cabin congested freeway	5	40
2	In-cabin urban canyon	3	40
3	In-cabin refueling	5	20
4	In-cabin underground garage	5	40
5	In-cabin toll plaza	3	40
6^a^	Roadway tunnel	5	40
7	Outdoor refueling	5	20
8	Sidewalk	3	40
8/9	Sidewalk/bus stop	3	40
10	Outdoor surface parking	3	40
11	Outdoor underground garage	5	40
12	Outdoor toll plaza	3	40
13 ^a^	In-cabin trailing high-emitting vehicles	5	40

^a^ME 13 was substituted for ME6 in Atlanta

Ambient CO and VOC data were retrieved from EPA’s Air Quality System (AQS) for the specific periods of the field study in each city and compared to the ME measurements. Only partial AQS VOC data were available for Atlanta and Chicago. Speciated summer-winter hourly GC/FID VOC data were available from two sites in Houston. All sites reporting CO and VOC data within each metropolitan area were averaged together for the purposes of intercity comparison since average CO and benzene concentrations did not show substantial intra-city spatial variation.

It must be recognized that the various microenvironments were selected with a goal to capture the 99th percentile exposure concentrations within each type of microenvironment, as prescribed by EPA and API. We also selected specific sampling times and locations with the greatest potential for higher exposures. These selections were based on considerations of various emission surrogates such as traffic counts, diurnal variations in average highway speeds, length of queues at toll plazas, and number of cars refueling or entering and exiting parking garages. Surrogates of dispersion included wind roses and diurnal variations in temperature. Measurements in microenvironments with unrestricted dispersion were made in the early morning or evening during calm conditions and minimal vertical mixing. In moderate wind conditions, we drove parallel to the prevailing wind to reduce the impact of cross winds. Thus, the ranges of exposure concentrations determined in this study are skewed toward the upper end of the distribution of exposure concentrations for each microenvironment.

### Apportionment of evaporative and exhaust emission contributions

Composition profiles for vehicle exhaust, liquid gasoline, and gasoline headspace vapor include many of the same species but have notable differences in the abundances of species that can be used to apportion tailpipe and evaporative emission source strengths. Although the use of MTBE as a gasoline additive has been phased out, it was a major component of gasoline in Houston during the study. The proportion of MTBE in exhaust is reduced during combustion relative to its proportion in the fuel. Conversely, benzene is enriched in exhaust relative to its proportion in the fuel due to toluene and xylene dealkylation during combustion. Consequently, MTBE/benzene ratios are lower in exhaust than in liquid fuel or headspace vapors. MTBE and benzene were measured in all microenvironments in Houston as well as in test fuels and vehicle exhaust samples. The fractional evaporative contribution, X, was estimated using following formula.

$$ X = \left({R}_{ME} - {R}_{EXH}\right)/\left({R}_{VAP} - {R}_{EXH}\right) $$

*R*_ME_ is the measured microenvironmental MTBE/benzene ratio, *R*_EXH_ is the exhaust ratio, and *R*_VAP_ is the evaporative vapor ratio—either of whole gasoline or headspace vapor depending upon type of evaporative emissions expected in a particular ME (e.g., whole liquid gasoline for hot soak, leaks, or spills and headspace vapors during refueling). Some MEs are influenced by a combination of whole gasoline and headspace vapor emissions.

The mass ratios of MTBE to benzene in vehicle exhaust, gasoline vapor, and liquid gasoline were determined from the evaporative and tailpipe emission tests conducted by Southwest Research for the 1993 Toyota Camry sedan and 1995 Ford F150 truck (Merritt 2005). Test fuel samples were subject to standard tests for Reid Vapor Pressure, distillation range, specific gravity, sulfur, benzene, hydrocarbon category (i.e., saturates, olefins, aromatics), oxygenated species (e.g., MTBE/EtOH), carbon weight percent, hydrogen weight percent, oxygen weight percent, and octane number. Gasoline headspace vapor compositions were predicted from the measured composition of liquid gasoline using the Kirchstetter et al. (1999) method with individual vapor pressures determined using the Wagner equation (Reid et al. 1987).

### Trailing vehicle experiment

For this study, SwRI determined the evaporative and tailpipe emissions of two test vehicles in the normal and malfunction modes using three test fuels. The test vehicles, a sedan and a full-sized V8 truck, were chosen within the 1993–1996 model years from vehicles with 90,000–110,000 odometer miles. The 1993 Toyota Camry sedan (2.2 L 4-cylinder engine) and 1995 Ford F150 Pickup truck (5.0 L V8 engine) were operated in an “as purchased” normal and malfunctioning (“high emitter”) mode, i.e., with the catalytic converter removed and NMHC emissions ≥ 2 g/mi as measured on the Federal Test Procedure (FTP) driving cycle. An additional calibrated manifold leak was needed to achieve ≥2 g/mi sedan emissions. SwRI determined dynamometer FTP emissions for each vehicle for all three fuels in the two emission modes (24 tests). Emission control components could be reproducibly adjusted to represent normal and reasonable high-end approximations (≥2 g/mi) of real-world exhaust emissions. Regulated exhaust emissions (THC, NMHC, CO, NOx), fuel economy, and specific VOCs (MTBE, EtOH, BTEX, 1,3-BD, HCHO) were determined in the dynamometer FTP tests. During hot-soak sealed housing for evaporated determination (SHED) tests, THC and specific VOCs (minus HCHO) were determined. Specific details of the emission tests are provided at Appendix F of Zielinska et al. (2009).

The two test vehicles were used as characterized sources (i.e., lead vehicles) ahead of an instrumented trailing vehicle (1996 Chrysler Minivan). Trailing vehicle in-cabin TVOC [ppbRAE-PID (photoionization detector)], CO (Langan T15), BTEX (Kore200MS), and HCHO (A-Ω) were continuously monitored and integrated VOC/NMHC (canister), HCHO/CH_3_CHO (DNPH cartridge), and EtOH (sorbent tube–EtOH fuel only) were collected. SPME BTEX samples were also collected every 10 min. Appendix B of Zielinska et al. 2009 describes the sampling and analytical methods in detail.

The trailing vehicle tests were conducted in an isolated location with minimal traffic south of San Antonio, TX, during summer 2002 and winter 2005 on county roads 462 and 2779 off IH 35 in the vicinity of Moore, Big Foot, and Jones Mound, Texas. The position of the trailing vehicle was recorded continuously by a Garmin 12XL GPS unit recording in UTM using NAD83/WGS84. The change in elevation over the 14.5-mi driving route was approximately 100 feet. The instrumented trailing vehicle was driven behind the test vehicles over a remote, paved, two-lane roadway loop for test periods up to 3 h. Initial measurements were made absent the lead test vehicles to established background levels. Trailing vehicle tests then implemented far, near, and passing scenarios at low (30 mph) and high speeds (60 mph). During “far” scenarios, “safe” vehicle spacing (defined as one car length—10 feet—for each 10 mph) was maintained. During the “near” scenario, the trailing vehicle tailgated the lead vehicle, following at a close distance deemed “safe” by the professional drivers under prevailing traffic and meteorological conditions. During the “passing” scenario, the trailing vehicle split its time between tailgating the lead vehicle and “passing,” immediately behind the lead vehicle but in the adjacent lane. A final idling test was conducted, while the trailing vehicle was parked on the road shoulder downwind and closely behind the parked idling lead vehicle. High (10 min) and low (10 min) ventilation conditions were used during all (including idling) tests.

## Results and discussion

Ambient pollutant concentrations are directly related to source emission rates in the ME and inversely related to source distance and the extent of dilution, itself a function of meteorology and any physical obstructions that inhibit dilution. Hourly values from air quality monitoring stations in urban areas typically represent neighborhood-scale exposure concentrations, while 20–40-min measures from MEs listed in Table 1 are intended to represent the high-end exposure concentrations during plausible “worst-case” conditions for these locations. These results should be compared to modeled high-end exposure concentrations or to samples collected with similar high-end sampling criteria and not to average ambient MSAT concentrations encountered by the general population.

### Comparison of high-end ME and ambient monitor values

**Fig. 1:**
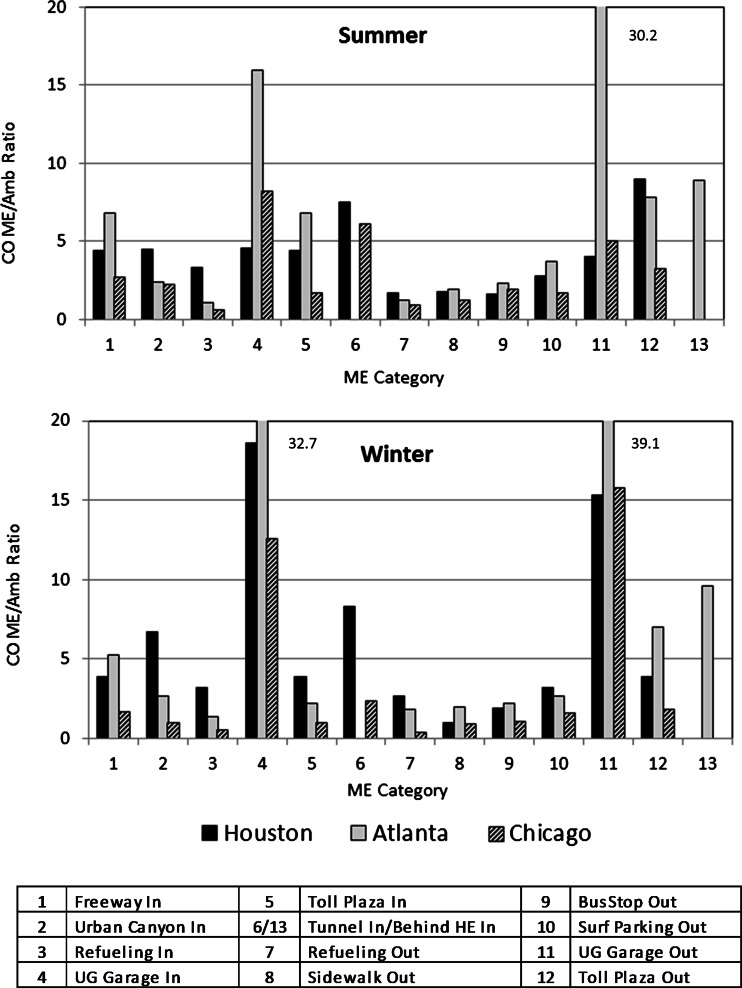
Median ratios of carbon monoxide measured in vehicle-dominated microenvironments in Houston, Atlanta, and Chicago in summer 2002 (*top*) and winter 2005 (*bottom*) relative to corresponding ambient measurements at the nearest air quality monitoring station (“proximity” factors). “In” and “Out” in the legend denote in-vehicle and outdoor microenvironments, respectively

CO is nonreactive and commonly measured year-round as a primary mobile source emission marker generally correlated with BTEX and 1,3-butadiene. Table 2 shows seasonal median and average ME/ambient ratios (proximity factors) and standard deviations (SD) from comparisons of time-integrated canister CO to corresponding hourly CO from ambient air monitoring stations in Houston, Atlanta, and Chicago. As shown in Fig. 1, the ratios are higher in MEs that are closer to moving vehicles, especially in underground garages where dispersion of pollutants is limited. Average summer in-cabin CO concentration ratios in congested freeway traffic range from 2 to 9 (2 to 5 in the winter) and were highest in Atlanta and lowest in Chicago. These seasonal and city-specific CO differences hold for most of the other MEs. A notable exception is the underground garage MEs (ME4, ME11) with higher winter ratios for all three cities, likely due to prolonged cold-start emissions at the lower temperatures. Garage size, ventilation, and vehicle activity patterns account for underground garage variability in CO levels. For example, the selected Atlanta high-end garage was smaller, less ventilated, and more in use than the garages in Houston or Chicago. While CO is a good exhaust emissions marker, correlation with evaporative emissions is poorer.

**Table 2:** Ratios (median; average ± SD) of the time-integrated high-end CO from canister samples and corresponding hourly CO from regional ambient air monitoring sites for Houston, Atlanta, and Chicago by season and microenvironment

Microenvironment	Houston	Atlanta	Chicago	
Summer				
1	Congested freeway, in-cabin	4.4; 4.6 ± 1.0	6.8; 9.1 ± 6.5	2.7; 2.6 ± 0.4
2	Urban canyon, in-cabin	4.5; 5.2 ± 2.5	2.4; 3.9 ± 2.7	2.2; 2.9 ± 1.4
3	Refueling, in-cabin	3.3; 3.6 ± 0.9	1.1; 1.4 ± 0.8	0.6; 0.7 ± 0.3
4	Underground garage, in-cabin	4.6; 8.4 ± 8.0	16.0; 16.9 ± 12.4	8.2; 7.9 ± 3.4
5	Toll plaza, in-cabin	4.4; 4.1 ± 1.0	6.8; 6.4 ± 1.8	1.7; 1.6 ± 0.5
6	Tunnel, in-cabin	7.5; 8.4 ± 1.7		6.1; 6.3 ± 1.5
7	Refueling, outdoor	1.7; 1.9 ± 0.8	1.2; 1.2 ± 0.5	0.9; 0.9 ± 0.3
8	Sidewalk	1.8; 1.9 ± 0.5	1.9; 1.9 ± 0.3	1.2; 1.1 ± 0.2
9	Sidewalk/bus stop	1.6; 1.5 ± 0.3	2.3; 2.4 ± 0.7	1.9; 1.6 ± 0.6
10	Surf parking, outdoor	2.8; 3.3 ± 1.2	3.7; 5.3 ± 3.6	1.7; 2.1 ± 1.3
11	Underground garage, outdoor	4.0; 8.6 ± 7.4	30.2; 26.8 ± 8.8	5.0; 3.9 ± 2.3
12	Toll plaza, outdoor	9.0; 7.6 ± 2.7	7.8; 6.5 ± 2.6	3.2; 3.2 ± 0.2
13	Following high emitter, in-cabin		8.9; 8.4 ± 3.9	
Winter				
1	Congested freeway, in-cabin	3.9; 4.0 ± 1.0	5.3; 5.4 ± 1.9	1.7; 1.7 ± 0.4
2	Urban canyon, in-cabin	6.7; 12.1 ± 10.9	2.7; 2.7 ± 0.6	1.0; 1.0 ± 0.5
3	Refueling, in-cabin	3.2; 4.1 ± 3.2	1.4; 1.3 ± 0.2	0.5; 0.5 ± 0.1
4	Underground garage, in-cabin	18.6; 21.4 ± 12.5	32.7; 38.8 ± 24.9	12.6; 12.6 ± 5.8
5	Toll plaza, in-cabin	3.9; 3.6 ± 0.6	2.2; 2.7 ± 1.4	1.0; 1.0 ± 0.1
6	Tunnel, in-cabin	8.3; 9.7 ± 5.2		2.4; 2.5 ± 0.4
7	Refueling, outdoor	2.7; 3.3 ± 2.5	1.8; 2.1 ± 0.9	0.4; 0.8 ± 0.9
8	Sidewalk	1.0; 1.3 ± 0.6	2.0; 1.9 ± 0.2	0.9; 0.9 ± 0.0
9	Sidewalk/bus stop	1.9; 1.8 ± 0.1	2.2; 1.9 ± 0.6	1.1; 1.1 ± 0.2
10	Surf parking, outdoor	3.2; 4.7 ± 2.9	2.7; 3.3 ± 2.0	1.6; 1.4 ± 0.5
11	Underground garage, outdoor	15.3; 17.1 ± 11.2	39.1; 32.9 ± 16.5	15.8; 12.4 ± 7.2
12	Toll plaza, outdoor	3.9; 6.2 ± 4.3	7.0; 7.4 ± 4.2	1.8; 1.8 ± 0.3
13	Following high emitter, in-cabin		9.6; 12.0 ± 5.6

**Fig. 2:**
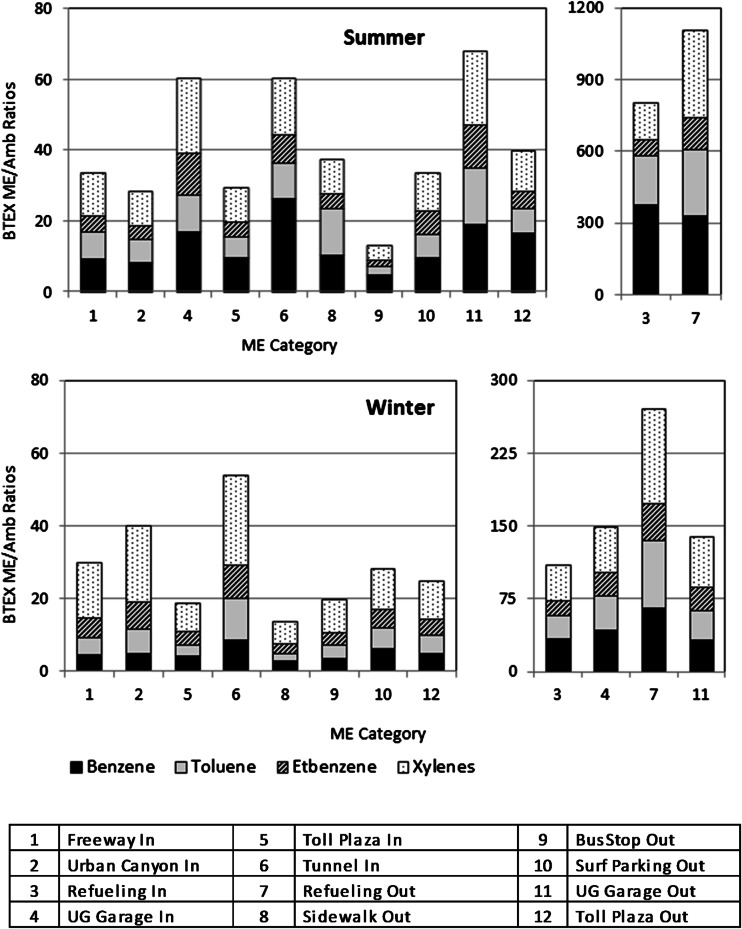
Median ratios of benzene, toluene, ethylbenzene, and xylenes (BTEX) measured in vehicle-dominated microenvironments in Houston in summer 2002 (*top*) and winter 2005 (*bottom*) relative to corresponding ambient measurements at the nearest air quality monitoring station (“proximity” factors). “In” and “Out” in the legend denote in-vehicle and outdoor microenvironments, respectively

Table 3 shows seasonal median and average Houston BTEX and 1,3-BD ME/monitor ratios (±SD). In contrast to CO, the ME/ambient ratios for BTEX are higher for refueling (ME3, ME7), especially during summer (Fig. 2). During the winter, the proximity factors for the refueling MEs are more comparable to those for underground garages (ME4, ME11). Since 1,3-BD is an exhaust-only species, the relative variation of ME/ambient ratios is similar to CO. Note, however, that 1,3-BD measures in MEs with high MTBE levels are compromised due to measurement method interference from isobutylene, a thermal decomposition product of MTBE occurring either in vehicle exhaust or during elevated temperature chromatographic analysis.

**Table 3:** Ratios (median; average ± SD) of BTEX and 1,3-BD from time-integrated canister samples and corresponding hourly automated GC data from the ambient air monitoring sites in Houston

Microenvironment	Benzene	Toluene	Etbenzene	*m*- and *p*-Xylene	*o*-Xylene	1,3-Butadiene	
Summer							
1	Congested freeway, in-cabin	9.2; 9.8 ± 3.8	7.7; 7.8 ± 1.9	4.5; 5.4 ± 1.8	5.3; 5.2 ± 0.6	7.0; 6.6 ± 1.5	8.3; 8.1 ± 3.6
2	Urban canyon, in-cabin	8.3; 7.4 ± 3.0	6.6; 6.8 ± 1.1	3.8; 5.6 ± 3.3	4.9; 5.6 ± 1.4	4.9; 5.8 ± 2.4	6.7; 7.3 ± 1.2
3	Refueling, in-cabin	373; 565 ± 791	205; 446 ± 666	71; 140 ± 188	75; 115 ± 136	78; 117 ± 146	bdl
4	Underground garage, in-cabin	16.8; 19.4 ± 13.9	10.6; 17.2 ± 15.0	11.9; 16.0 ± 13.7	9.9; 14.2 ± 12.8	11.1; 17.1 ± 17.0	22.6; 50.0 ± 66.4
5	Toll plaza, in-cabin	9.6; 9.1 ± 4.4	5.9; 7.8 ± 3.6	4.2; 6.8 ± 4.9	4.4; 6.2 ± 3.4	5.2; 7.1 ± 3.8	5.4; 7.8 ± 5.2
6	Tunnel, in-cabin	26.3; 25.9 ± 14.1	10.1; 12.4 ± 6.8	8.1; 7.8 ± 2.9	7.1; 7.5 ± 2.7	8.8; 8.9 ± 2.7	26.7; 37.9 ± 20.0
7	Refueling, outdoor	328; 1311 ± 1811	279; 974 ± 1356	133; 335 ± 479	206; 219 ± 227	159; 197 ± 197	bdl
8	Sidewalk	10.3; 15.4 ± 10.6	13.1; 10.6 ± 4.4	4.4; 12.5 ± 15.3	4.5; 11.9 ± 14.0	5.2; 14.2 ± 17.1	6.5; 5.8 ± 2.4
9	Sidewalk/bus stop	4.9; 4.7 ± 2.0	2.4; 2.9 ± 1.3	1.8; 1.8 ± 0.4	1.9; 1.9 ± 0.5	2.0; 2.3 ± 0.6	1.7; 2.0 ± 1.3
10	Surf parking, outdoor	9.6; 11.5 ± 5.5	6.7; 8.3 ± 2.8	6.7; 7.4 ± 2.1	5.3; 6.7 ± 3.1	5.3; 7.0 ± 3.2	5.0; 5.5 ± 1.9
11	Underground garage, outdoor	19.1; 16.7 ± 5.3	16.0; 15.7 ± 7.2	11.9; 13.8 ± 7.2	9.2; 11.1 ± 7.2	11.7; 14.4 ± 9.6	46.0; 39.6 ± 29.5
12	Toll plaza, outdoor	16.6; 14.7 ± 8.5	7.1; 11.3 ± 10.2	4.8; 9.2 ± 8.3	5.4; 8.5 ± 7.6	6.1; 11.0 ± 10.6	7.6; 13.4 ± 13.1
Winter							
1	Congested freeway, in-cabin	4.2; 4.6 ± 1.6	4.9; 6.4 ± 3.7	5.4; 6.4 ± 3.0	7.1; 8.8 ± 5.2	8.1; 9.7 ± 5.1	0.32; 0.40^a^
2	Urban canyon, in-cabin	4.6; 17.0 ± 21.7	6.7; 22.7 ± 27.7	7.6; 22.1 ± 26.4	9.8; 31.1 ± 36.9	11.2; 26.7 ± 28.9	bdl
3	Refueling, in-cabin	34; 35 ± 25	24; 36 ± 27	15; 23 ± 18	18; 25 ± 19	19; 24 ± 16	bdl
4	Underground garage, in-cabin	41.8; 37.2 ± 15.8	35.8; 32.3 ± 12.9	24.3; 24.0 ± 9.4	22.8; 23.2 ± 8.5	23.9; 25.0 ± 9.7	bdl
5	Toll plaza, in-cabin	4.1; 3.5 ± 1.7	2.9; 3.7 ± 1.5	3.7; 4.3 ± 1.7	3.6; 4.6 ± 2.0	4.3; 5.3 ± 2.1	bdl
6	Tunnel, in-cabin	8.3; 8.5 ± 6.7	11.6; 9.0 ± 6.9	9.2; 7.0 ± 4.5	11.8; 8.9 ± 6.4	12.7; 9.9 ± 7.2	1.52; 2.29^a^
7	Refueling, outdoor	65; 160 ± 148	70; 119 ± 92	38; 49 ± 39	50; 56 ± 54	47; 45 ± 34	bdl
8	Sidewalk	2.6; 2.8 ± 0.8	2.2; 2.6 ± 1.0	2.5; 2.7 ± 0.7	2.9; 3.6 ± 1.7	3.3; 3.7 ± 1.3	0.02; 0.16^a^
9	Sidewalk/bus stop	3.3; 3.3 ± 0.3	3.7; 3.6 ± 0.3	3.3; 3.5 ± 1.1	4.5; 4.2 ± 0.7	4.6; 4.3 ± 0.8	bdl
10	Surf parking, outdoor	6.0; 4.3 ± 3.0	5.8; 7.5 ± 6.4	5.0; 5.7 ± 2.9	5.3; 6.1 ± 2.1	5.8; 6.0 ± 2.5	bdl
11	Underground garage, outdoor	31.8; 28.3 ± 16.5	31.0; 25.0 ± 13.0	23.9; 20.8 ± 11.3	24.1; 21.9 ± 11.1	27.3; 22.9 ± 10.7	0.58; 0.62^a^
12	Toll plaza, outdoor	4.6; 4.8 ± 0.7	5.1; 4.5 ± 1.4	4.4; 4.5 ± 1.8	5.1; 4.9 ± 1.6	5.3; 5.4 ± 1.9	bdl

^a^Only two valid samples

*bdl* below detectable limit

The HAPEM4 factors may be elevated compared to 2002–2005 conditions since they are based on measurements taken before 1991 when benzene fuel levels and roadway fleet emissions were higher. HAPEM5 proximity factors may also need to be adjusted since they were based in part on a 1998 scoping study (Rodes et al. 1998) where measurements “highlighted trailing behind heavy duty diesel vehicles and diesel city buses when possible.” Houston ME/ambient ratio comparisons also generally exceed the other HAPEM factors listed in Table 4. Refueling ratios (ME3, ME7) for benzene were higher than the corresponding HAPEM factors also as expected with inclusion of scripted fuel spillage, and concentrations of exhaust components such as CO and 1,3-BD were enhanced under reduced ventilation situations.

**Table 4:** Houston benzene median ME/ambient ratio comparison with HAPEM benzene ME factors

Microenvironment	Summer	Winter	HAPEM4 PROX	HAPEM5 PROX distribution (mode; median)		HAPEM5 PROX range
1	Congested freeway, in-cabin	9.2	4.2	5.2, 6.9*	Triangular 1.9; 4.9	0–14.4
2	Urban canyon, in-cabin	8.3	4.6	4.4	Triangular 1.9; 4.9	0–14.4
3	Refueling, in-cabin	372.8	34.4	4.4	Triangular 1.6; 2.7	0–7.1
4	Underground garage, in-cabin	16.8	41.8	1	Triangular 1.6; 2.7	0–7.1
5	Toll plaza, in-cabin	9.6	4.1	4.4	Triangular 1.9; 4.9	0–14.4
6	Tunnel, in-cabin	26.3	8.3	4.4	Triangular 1.9; 4.9	0–14.4
7	Refueling, outdoor	327.7	65.2	4.4	Triangular 1.6; 2.7	0–7.1
8	Sidewalk	10.3	2.6	4.4	Triangular 1.6; 2.7	0–7.1
9	Sidewalk/bus stop	4.9	3.3	4.4	Triangular 1.6; 2.7	0–7.1
10	Surf parking, outdoor	9.6	6.0	4.4	Triangular 1.6; 2.7	0–7.1
11	Underground garage, outdoor	19.1	31.8	1	Triangular 1.6; 2.7	0–7.1
12	Toll plaza, outdoor	16.6	4.6	4.4	Triangular 1.6; 2.7	0–7.1

### Apportionment of exhaust and evaporative emissions exposure concentrations

The measured MTBE/benzene ratios are summarized in Table 5 for the Houston MEs. Exposure concentrations at the two refueling MEs (ME3 & ME7) were dominated by evaporative emissions as expected, with MTBE/benzene ratios of 20 to 30, whereas ratios for all other MEs were between 1 and 4. The exhaust, headspace, and liquid fuel MTBE/benzene ratios measured in this and other recent studies are shown in Table 6. MTBE/benzene exhaust ratios among the FTP dynamometer tests average about 1, whereas the tunnel ratios were nearer to 2, possibly due to the added running loss emissions (evaporative emissions during vehicle operation) in tunnels. The MTBE/benzene ratios in liquid gasoline and headspace vapor are similar, averaging 15–20. Ratios for the SwRI FTP tests are comparable with calculated headspace compositions and values from earlier listed studies.

**Table 5:** Mass ratios (median; average ± SD) of MTBE to benzene in Houston by ME

Microenvironment		Summer	Winter
1	Congested freeway, in-cabin	1.6; 1.7 ± 0.5	2.7; 2.9 ± 1.3
2	Urban canyon, in-cabin	0.9; 1.1 ± 0.4	2.2; 2.6 ± 0.8
3	Refueling, in-cabin	19.8; 24.9 ± 12.2	40.4; 42.0 ± 21.3
4	Underground garage, in-cabin	3.3; 3.2 ± 1.5	2.4; 2.7 ± 1.5
5	Toll plaza, in-cabin	2.2; 2.5 ± 0.8	3.6; 3.0 ± 1.1
6	Tunnel, in-cabin	2.7; 2.7 ± 0.4	2.7; 5.3 ± 6.6
7	Refueling, outdoor	23.1; 28.8 ± 12.0	50.9; 55.8 ± 26.8
8	Sidewalk	2.3; 2.3 ± 0.2	1.1; 1.2 ± 0.1
9	Sidewalk/bus stop	2.3; 2.9 ± 1.1	1.4; 1.3 ± 0.2
10	Surf parking, outdoor	1.9; 1.9 ± 0.4	4.8; 6.9 ± 6.6
11	Underground garage, outdoor	2.7; 2.6 ± 0.4	3.0; 3.3 ± 1.5
12	Toll plaza, outdoor	1.5; 2.5 ± 1.8	2.8; 2.8 ± 1.2

**Table 6:** Mass ratios of MTBE to benzene in vehicle exhaust, gasoline vapor and liquid gasoline

Test set	Fuel	Year	Average	Standard deviation	References
Ratios in LDGV exhaust					
CRPAQS/GDS dyno exhaust	Los Angeles	2001	0.62	1.35	Fitz et al. 2003
CRPAQS/GDS dyno warm-starts	Los Angeles	2001	0.43	1.43	Fitz et al. 2003
CRPAQS/GDS dyno high emitter	Los Angeles	2001	1.11	3.17	Fitz et al. 2003
SWRI FTP—summer	Houston	2004	0.44	0.24	Merritt 2005
SWRI FTP—winter	Houston	2005	1.4	0.55	Merritt 2005
SWRI FTP—normal	Houston	2004, 2005	0.6	0.45	Merritt 2005
SWRI FTP—malfunction	Houston	2004, 2005	1.24	0.71	Merritt 2005
Ratios in tunnel and roadway samples					
Weekend ozone study—on road	Los Angeles	2000	2.96	1.03	Fujita et al. 2003a, b
LA tunnels—corrected for running loss	Los Angeles	1995, 1996	0.32	0.99	Fujita et al. 2003b
LA tunnels—uncorrected	Los Angeles	1995, 1996	1.69	0.54	Fujita et al. 2003b
S211(b) Study Houston tunnel (ME6)	Houston	2004, 2005	3.36	1.35	This Study
Ratios in gasoline vapor					
LA vapor	Los Angeles	1995	16.63		Fujita et al. 2003b
Weekend ozone study vapor	Los Angeles	2000	44.98		Fujita et al. 2003a, b
SWRI SHED—malfunction	Houston	2004, 2005	20.52	29.2	Merritt 2005
SWRI SHED—malfunction outlier removed	Houston	2004, 2005	5.93	1.6	Merritt 2005
S211(b) Study estimated from fuel—summer	Houston	2004	32.8		Merritt 2005
S211(b) Study estimated from fuel—winter	Houston	2005	43.9		Merritt 2005
Ratios in liquid gasoline					
LA gasoline—1995 RFG	Los Angeles	1995	11.7		Fujita et al. 2003b
Weekend ozone study	Los Angeles	2000	19.56	5.23	Fujita et al. 2003a, b
Gas diesel split study	Los Angeles	2001	17.15	5.49	Gabele 2003
S211(b) Study gasoline—summer	Houston	2004	13.28		Merritt 2005
S211(b) Study gasoline—winter	Houston	2004	17.87		Merritt 2005

**Fig. 3:**
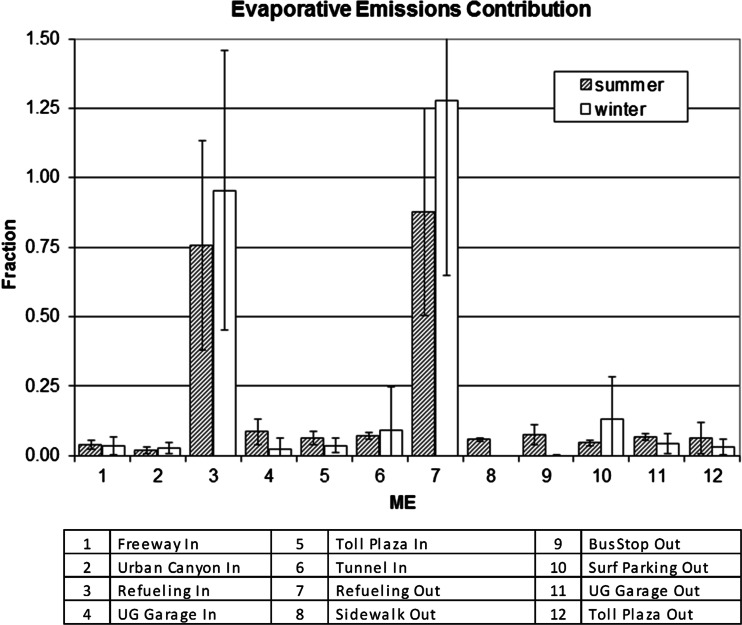
Fractional contributions of evaporative emissions to total motor vehicle emissions in vehicle-dominated microenvironments in Houston in summer 2002 and winter 2005

The estimated fractional contributions of evaporative emissions to total motor vehicles emissions for the various Houston MEs, using the above method, are summarized in Fig. 3. Attributions of evaporative emissions to measured exposure concentrations during refueling (ME3, ME7) approach 100 % within the uncertainties associated with the measurements and method. ME3 simulates open window full-service in-cabin refueling exposure concentrations with ME7 representing self-serve outdoor exposure concentrations. All other MEs are dominated by tailpipe emissions with evaporative emission contributions below 10 %.

Evaporative emissions were the substantial components of high-end ME exposure concentrations only during vehicle refueling operations. Since refueling MEs had the highest relative levels of these components, they constituted the peak exposure concentrations, although overall the average population time spent in these high-end MEs is likely the shortest. The contributions of evaporative emissions for all other MEs were typically about 5 % of the total measured concentrations. Results were similar for both seasons, although evaporative contributions during refueling were marginally less during summer, possibly due to more rapid dispersion of vapors and evaporation of fuel spilled during each refueling test at higher summer temperatures. Refueling emissions released from pressurized fuel systems and spilled fuels appeared to be the primary source of peak evaporative exposure concentrations. The lack of a seasonal variability in the evaporative contribution was also consistent with this supposition. Although it was not feasible to try to distinguish further between liquid and headspace vapor contributions by the method used, the extremely low MTBE/benzene ratios observed for the roadway, sidewalk, and parking MEs strongly suggest that the impact from leaking liquid gasoline emissions was minor.

### Effect of ventilation condition and proximity on in-vehicle exposure concentrations of a trailing vehicle

**Fig. 4:**
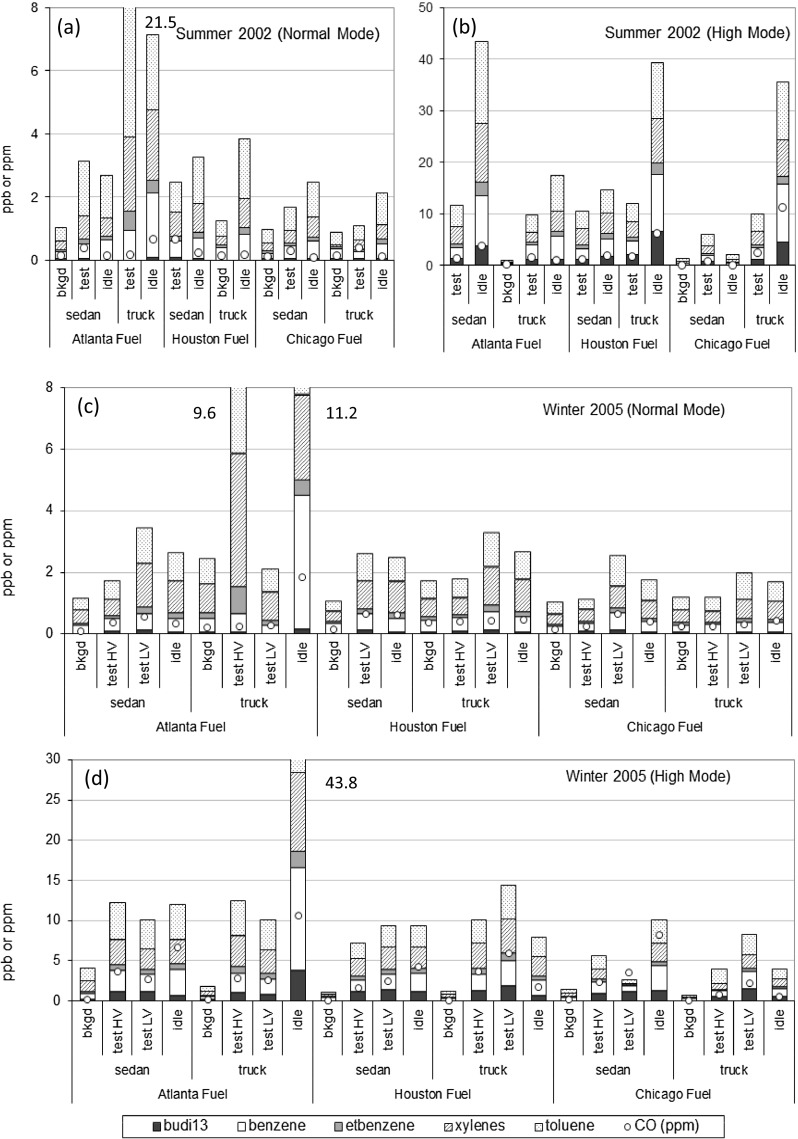
Concentrations of 1,3-BD, BTEX and CO as measured in summer 2002 [normal mode (a) and high mode (b)] and winter 2005 [normal mode (c) and high mode (d)] inside the trailing vehicle cabin under high (HV, window and vent open and fan on) and low (LV, windows closed and vent on recirculate) ventilation while following the lead vehicle (either sedan or truck) operated in normal or high emission mode

The trailing vehicle experiment was conducted to relate in-vehicle pollutant levels to the following variables: background pollutant concentrations, exhaust emission rate of the lead vehicle, ventilation condition of the instrumented trailing vehicle, and proximity and speed of the lead and trailing vehicles. A rural test site with very little traffic was chosen to minimize nonlead vehicle influences on the in-cabin pollutant levels of the trailing vehicle. Figure 4 shows the MTBE, BTEX, and CO concentrations for each test segment during summer 2002 and winter 2005.

Background levels of the target species were low and consistent from summer to winter. Trailing vehicle cabin values were larger than background values. Idle test sample values were on average twice those encountered in driving tests, suggesting that proximity and proximity duration may impact in-cabin trailing vehicle concentrations. Average in-cabin levels were not similar for the two lead vehicles in normal mode or using conventional versus oxygenated fuels. Similarly, the season had no strong effect upon the trailing vehicle concentrations.

Trailing vehicle tests indicated that the largest impact on in-cabin values came from the emissions mode of the leading vehicle. Averaging over the fuels and vehicles, the high emitter mode resulted in much higher trailing vehicle in-cabin values than the normal emitter mode, on average 2.2 times higher; except for HCHO which was relatively unchanged from background. Trailing vehicle ventilation status also affected in-cabin values. Although mean canister (integrated) in-cabin values were similar under high (window and vent open and fan on) and low (windows closed and vent on recirculate) ventilation conditions, the range of concentrations observed by continuous PID and CO monitors was much larger under high ventilation. This may be rationalized as the vehicle moving into and out of the exhaust plume of the leading vehicle with in-cabin values changing rapidly under high ventilation, whereas under low ventilation, in-cabin concentrations trapped as the vents were closed stayed relatively constant during the remainder of low ventilation conditions. During maximum ventilation, the in-vehicle pollutant concentrations were comparable to, and tracked, on-road concentrations. Closed ventilation tends to cause a “memory effect” for gaseous pollutants in which the in-vehicle air retains the initial pollutant concentrations. Other studies have shown that PM_2.5_ and BC concentrations are lower inside the vehicle than on-road during minimum ventilation and decreases with time when in-vehicle air is recirculated through the in-cabin filter (Fujita et al. 2014).

## Conclusions

This study provides real-world measurements of gaseous pollutant in vehicle-dominated microenvironments and estimates of proximity factors that can be used to evaluate exposure model results. The high-end MEs in close proximity to active vehicle engines have enhanced CO relative to ambient levels with ME/ambient ratios approaching 40 where ventilation is limited, as in underground garages. Average proximity factor in congested freeway traffic ranges from 2 to 9 with lower winter ratios. ME locations that are less proximate to operating vehicles such as gas stations and urban sidewalks have CO/ambient ratios of 0.5–2. In contrast to CO, the ME/ambient ratios for BTEX are higher, especially for refueling MEs during summer reflecting the greater rates of evaporative emissions compared to the cooler winter season. With the exception of vehicle refueling operations, the results indicate that evaporative emissions are a minor component of high-end MSAT exposure concentrations.
